# Burden of Placental Malaria among Pregnant Women Who Use or Do Not Use Intermittent Preventive Treatment at Mulago Hospital, Kampala

**DOI:** 10.1155/2016/1839795

**Published:** 2016-12-13

**Authors:** Charles Okot Odongo, Michael Odida, Henry Wabinga, Celestino Obua, Josaphat Byamugisha

**Affiliations:** ^1^Department of Pharmacology and Therapeutics, School of Biomedical Sciences, Makerere University College of Health Sciences, P.O. Box 7072, Upper Mulago Hill Road, Kampala, Uganda; ^2^Department of Pharmacology and Therapeutics, Faculty of Medicine, Gulu University, P.O. Box 166, Gulu, Uganda; ^3^Department of Pathology, School of Medicine, Makerere University College of Health Sciences, P.O. Box 7062, Kampala, Uganda; ^4^Mbarara University of Science and Technology, P.O. Box 1410, Mbarara, Uganda; ^5^Department of Obstetrics and Gynaecology, School of Medicine, College of Health Sciences, Mulago Hospital Complex, P.O. Box 7062, Kampala, Uganda

## Abstract

Intermittent preventive treatment of malaria in pregnancy with sulphadoxine-pyrimethamine (SP-IPTp) is widely used to reduce the incidence of adverse pregnancy outcomes. As a monitor for continued effectiveness of this intervention amidst SP resistance, we aimed to assess malaria burden among pregnant women who use or do not use SP-IPTp. In a descriptive cohort study at Mulago Hospital, Kampala, 87 women who received two supervised doses of SP-IPTp were followed up until delivery. Controls were pregnant women presenting in early labour without history of SP-IPTp. Histopathological investigation for placental malaria (PM) was performed using the Bulmer classification criterion. Thirty-eight of the 87 women returned for delivery and 33 placentas were successfully collected and processed along with 33 placentas from SP nonusers. Overall, 12% (4/33) of the users had evidence of PM compared to 48% (16/33) of nonusers. Among nonusers, 17/33, 8/33, 2/33, and 6/33 had no placental infection, active infection, active-chronic infection, and past-chronic infection, respectively. Among users, respective proportions were 29/33, 2/33, 0/33, and 2/33. No difference in birth weights was apparent between the two groups, probably due to a higher proportion of infections occurring later in pregnancy. Histological evidence here suggests that SP continues to offer substantial benefit as IPTp.

## 1. Background

The increased attractiveness to infectious mosquitoes [1] coupled with transient depression in cell-mediated immunity [2] increases pregnant women's susceptibility to malaria and its adverse birth-related outcomes. Each year, more than 30 million pregnancies at risk of malaria occur in sub-Saharan Africa [3]. Malaria in pregnancy (MIP) is therefore of major public health concern. The adverse effects of MIP vary geographically by malaria transmission intensities. In high transmission settings where repeated exposure to infectious mosquito bites is common, most people acquire partial protective immunity by early adulthood. However, pregnant women in these settings remain comparatively more susceptible to malaria than their nonpregnant counterparts as both cellular and humoral immune responses to* Plasmodium *antigens are depressed [2, 4]. In such settings, MIP is mainly characterised by asymptomatic parasitemia often with sequestration of parasites in the placenta with maternal anaemia and low birth weight (LBW) as the principal adverse outcomes [5, 6]. In areas of low transmission, most women attain reproductive age with relatively little acquired immunity to malaria. Here, MIP is associated with severe clinical illness often with increased risks of miscarriages, still births, LBW, or even maternal death [5].

The pathophysiological processes preceding the adverse foetal consequences of MIP are mainly initiated by accumulation of parasitized red blood cells (pRBCs) in placental intervillous spaces. These pRBCs express parasite-encoded surface antigens (e.g., Pf EMP-1) that enable their sequestration to host receptors such as chondroitin sulphate A and hyaluronic acid [7]. This is followed by infiltration of inflammatory cells and release of proinflammatory mediators [8] and finally by syncytiotrophoblastic necrosis and the deposition of fibrinoid material [9]. Placental malaria (PM) is therefore a major manifestation of MIP and often occurs without peripheral symptoms [6]. Previous studies estimating the burden of PM in sub-Saharan Africa have yielded variable findings. In one review, prevalence estimates were in the range 9.5–37% using placental blood smears, 41–43% using rapid antigen tests, 51–59% using PCR, and 55–75% using placental histology [6]. While this may reflect true underlying differences in disease burden by study sites, these differences have been partly attributed to sensitivity differences in the tools used. However, despite the fact that placental blood smear was the least sensitive method, most previous studies have used it, probably due to its ease of application [6]. It therefore appears that, more often than not, previous studies have underestimated the true burden of PM and thus malaria burden in pregnancy. Nonetheless, it was estimated in 2007 that at least 25% of African pregnancies were afflicted by malaria at the time of delivery [5].

In Kampala district, an area of moderate malaria transmission, data from Mulago Hospital collected ten years earlier showed that 14% of deliveries had evidence of malaria infection [10]. The country has since witnessed a sustained malaria control campaign implemented by the government in collaboration with development partners. Going by recent reports, remarkable success has been achieved [11]. Key elements of this campaign have included the introduction of artemisinin-based combination therapies (ACTs) for effective case management, promotion and mass distribution of insecticide-treated bed nets (ITNs), and increased public education to improve treatment-seeking and prevention practices. Whereas these interventions have targeted the population at large, intermittent preventive treatment with sulfadoxine-pyrimethamine (SP-IPTp) has been particularly implemented as an additional measure to prevent MIP [12]. Yet, recent times have seen increasing concerns about the effectiveness of SP-IPTp both locally [13–15] and globally [16–18]. These concerns have been largely driven by the rising prevalence of higher-level SP resistance mutations. To this end, recent studies from areas with high prevalence of resistance mutations have reported reduced efficacy of SP-IPTp [14, 15, 19, 20]. However, such findings in most cases are hampered by the exclusive reliance on self-reports and antenatal records to ascertain SP use. Because unsupervised SP-IPTp administration is a common occurrence in many resource-limited settings, we have previously argued that self-reports and ANC records may not necessarily translate into SP intake compliance [21, 22]. Moreover, where intake was ascertainable, studies have continued to show effectiveness of SP-IPTp [13, 23–29]. Consequently, some authors have questioned the usefulness of molecular markers in monitoring SP-IPTp effectiveness [25, 27, 30]. More recently however, the* Pfdhps* A581G mutation (a proxy for higher-level resistance) has emerged as a better marker for SP-IPTp effectiveness [31, 32]. Prevalence levels exceeding 10% have been found to correlate with SP-IPTp failure [33]. In Kampala district where prevalence of the quintuple mutation is high but that of the A581G mutation is thought to be low, this study aimed to assess the relevance of continued SP-IPTp use. Placental histology, a robust and sensitive method, was used to examine malaria burden among pregnant women who received or did not receive intermittent preventive treatment.

## 2. Materials and Methods

### 2.1. Study Design

This was a descriptive cohort study conducted among pregnant women at Mulago Hospital between September and November 2014. The exposure arm of this cohort was part of a larger population pharmacokinetic study in which 87 pregnant women received two supervised doses of SP-IPTp and were followed up until delivery. For the control arm, pregnant women presenting in early labour, with no history of SP use, were consecutively identified and invited to participate in the study. These women were required to donate venous blood samples for a retrospective assessment of SP exposure covering the previous 2-3 months. All women enrolled were required to donate their placentas for histological investigation.

### 2.2. Study Site and Study Population

Mulago Hospital is Uganda's national referral facility located in Kampala, the capital and commercial centre. In addition to a wide range of specialized inpatient care, the hospital runs a range of outpatient clinics on week days. Most outpatients come from the urban and periurban communities of Kampala and the neighbouring districts of Mukono and Wakiso. These areas are mainly inhabited by the ethnic Bantu, particularly the Baganda. Over time, however, other Ugandan ethnic communities have settled here, constituting a significant minority. Most outpatients tend to be low- and middle-income earners who prefer to utilize the hospital's free services. Malaria is endemic in 95% of Uganda's geographic area with the remaining 5% (mainly highland areas with altitudes greater than 1,600 m) subject to low and seasonal transmission. Kampala is situated close to the equator at altitudes ranging from 1,300 to 1,500 m above the sea level and experiences a tropical climate with two rainy seasons (March to May and September to November). The last twenty years have witnessed a steady increase in Kampala's urbanization, a change that has possibly contributed to a reduction in malaria burden. Recent figures on transmission intensity are hard to come by, but prevalence of malaria among children aged 0–59 months in the greater Kampala region has recently been estimated at 5.5%, down from 12.1% in 2009 [11]. While the prevalence of the* dhfr/dhps* quintuple mutations is generally high for Kampala (>80%), there are no reliable estimates for the prevalence of the* Pfdhps* A581G mutation. However, recent estimates from the neighbouring Mukono district placed the prevalence at 3.9% among pregnant women presenting for ANC [13].

### 2.3. Study Setting

Hospital records show that approximately 1,000 outpatients are seen daily, with 25% being pregnant women seeking obstetric care at the two ANC clinics located at Old and New Mulago. These clinics offer general and referral antenatal services, respectively. All pregnant women seeking care initially present to the general ANC clinic run by a team of experienced midwives who offer a standard package of routine ANC services. Women who require the opinion of an obstetrician or those considered to have high risk pregnancies are referred to the New Mulago ANC clinic. At term, regular attendees of the general clinic, especially those deemed to have undergone a normal pregnancy, are directed for delivery in Ward 14, a labour unit located within the Old Mulago complex and also run by a team of experienced midwives. Women with high risk pregnancies as well as those presenting randomly for the first time while in labour are referred for delivery in Ward 5C located within New Mulago complex. Here, unlike the setting at Old Mulago, additional services such as caesarean sections, blood transfusions, and special care facilities for premature babies are readily available.

### 2.4. Study Procedure and Enrollment Criteria

SP-IPTp users were enrolled from the general ANC clinic at Old Mulago while in the second trimester of gestation (between 16 and 20 weeks). The women received two doses of SP (one in each of the second and third trimesters) administered under the direct supervision of a midwife in accordance with national guidelines for SP-IPTp at that time [12]. For ethical reasons, SP nonusers were only recruited in early labour from among pregnant women presenting to Ward 5C for delivery. To be eligible for enrollment, a woman had to have reported nonuse of SP-IPTp during the present pregnancy. As such claims may lack credibility for several reasons [34], each woman was required to provide a venous blood sample (4 mL) for detection of sulphadoxine (SDX). This requirement allowed for a retrospective assessment of SP exposure over the previous 2-3 months. Upon delivery, placentas from all participants were saved for histological investigation. The exclusion criteria included women with known chronic conditions such as HIV or cardiovascular, renal, metabolic, or sickle cell diseases. Women who developed pregnancy-related complications such as preeclampsia or antepartum haemorrhage were also excluded.

### 2.5. Ethical Statement

All participants provided written informed consent prior to enrollment. The study protocol received ethical approval from the hospital's research ethics committee (reference number MREC 397) as well as the Uganda National Council for Science and Technology (reference number HS 1277). All study procedures were performed in accordance with ethical guidelines of the Helsinki declaration (1964) and its amendments as well as the International Conference on Harmonization (ICH) guidelines on Good Clinical Practice.

### 2.6. Data Collection

Within minutes of delivery, the placenta was placed in a labelled container fully immersed in 10% neutral-buffered formalin before transportation to the pathology laboratory for storage and further processing. Whole placentas were initially stored for 24 hours in order to firm up the tissue in preparation for biopsy and definitive storage. For each placenta, a 4 × 4 cm biopsy was cut paracentrically involving the entire thickness of the placenta. This biopsy was placed in another container with 10% neutral-buffered formalin and fixed for 24 hours before being transferred to another container with 70% ethanol where it was stored for 2-3 months awaiting histological preparation and assessment. Within hours of delivery, the attending midwife collected brief demographic and obstetric data using a standardized questionnaire. Items covered included maternal and gestational age, birth weight, sex of baby, history of bed-net use, and physical address of residence.

### 2.7. Placental Histological Examination

All the histological investigation was done at the Department of Pathology, College of Health Sciences, Makerere University. Placental tissues were processed and embedded in paraffin wax using standard techniques. Paraffin sections, 5 *μ*m thick, were stained with hematoxylin and eosin (H&E) and Giemsa's stain. Sections were examined under light microscope by two histopathologists blinded to participant identities. Each slide was scored for footprints of placental malaria based on the classification criteria proposed by Bulmer et al. [35]. This classification is based on the presence or absence of parasites or malaria pigment and has the ability to provide temporal information on malaria history for more than half the pregnancy. Briefly, the classification may be summarized as follows:* Category 0* (placenta not infected; here, there is no evidence of parasites or malaria pigment observed);* Category 1* (active infection; parasites seen in maternal erythrocytes in the intervillous space or pigment in erythrocytes and monocytes in the intervillous space; there is no pigment in fibrin or cells within fibrin);* Category 2* (active-chronic infection; parasites in maternal erythrocytes in intervillous space or pigment present in erythrocytes and circulating monocytes within the intervillous space; in addition, there is pigment in fibrin or cells within fibrin and/or chorionic villous syncytiotrophoblast or stroma);* Category 3* (past-chronic infection; parasites not present, pigment confined to fibrin or cells within fibrin). All cases of discrepancy in histologic classification were resolved by consensus between the examiners.

### 2.8. The SP Bioanalytical Assay

All bioanalytical procedures for SDX were performed at the Department of Pharmacology and Therapeutics, College of Health Sciences, Makerere University. Venous blood samples collected from participants reporting nonuse of SP were centrifuged at 3000 revolutions per minute for 10 minutes. Two millilitres of plasma was transferred into cryotubes and stored at −70 degrees centigrade until analysis. Plasma was assayed for the presence of SDX using a high performance liquid chromatography-UV method as previously described by Bergqvist et al. [36]. Detection of SDX in plasma was considered evidence of prior SP intake. Therefore, any positive finding served to discredit the claim of SP nonuse while a negative finding corroborated such claims. With a limit of detection (LOD) as low as 7 *μ*mol/L, it was calculated that SDX would be detectable in plasma for at least 10 weeks from the time of intake (assuming an average *C*_max_ of 300 *μ*mol/L and a half-life of 13–15 days) [37]. As accurate quantification of plasma SDX levels was not of paramount interest, all assay procedures were performed once for each participant sample.

## 3. Results

Of the 87 women for whom supervised administration of SP-IPTp was done, 38 returned for delivery at Ward 14 and, from these, 33 placentas were successfully collected and processed for histological examination. To match the number of placentas in the exposure with the control arm, 33 women were also enrolled from the control group presenting to Ward 5C. None of the blood samples from the controls contained detectable levels of SDX. As shown in Table 1, participants in both groups appeared comparable on most baseline characteristics such as age, history of bed-net use, gestational age at delivery, birth weight, and baby's sex distribution. While 94% of the SP users resided within Kampala and the neighbouring districts of Mukono and Wakiso, 79% of the control group were from these areas. Therefore, 21% came from remote districts such as Masaka and Iganga.

Table 2 shows the results of histologic investigations on placentae. The burden of placental malaria was lower among SP users (4/33) compared to SP nonusers (16/33). Among SP nonusers with a positive malaria footprint, most (8/16) had evidence of active infection at time of delivery.

## 4. Discussion

In this limited sample of deliveries at Mulago Hospital in Kampala, women who do not use SP-IPTp seem to experience a higher burden of placental malaria compared to users. This finding suggests that SP may still be beneficial in preventing placental malaria and its associated adverse outcomes. This finding is consistent with clinical outcomes from several recent studies that assessed the efficacy of SP-IPTp in settings with a high prevalence of SP resistance mutations [13, 23, 24, 26–28]. SP-IPTp works by the clearance of asymptomatic parasitemia followed by posttreatment prophylaxis (PTP) [38]. The latter effect occurs over a variable length of time depending on drug dose, pharmacokinetic properties, and level of parasite resistance. As the first two factors are relatively constant, it has been suggested that increasing prevalence of higher-level resistance would correlate with reduction in the duration of PTP [38]. Notwithstanding such changes, the present results suggest that SP retains significant antimalarial activity in the context of IPTp. This finding is consistent with previous reviews that have shown considerable effectiveness of SP-IPTp even in areas with >90% presence of the* Pfdhps* K540E mutation (a proxy marker for the presence of quintuple mutation) [30, 39]. These observations suggest that additional factors may be critical in determining overall effectiveness of SP-IPTp. Francis et al. have previously shown that acquired antimalarial immunity was a better predictor of malaria treatment outcomes when compared with molecular markers of SP resistance [40]. It therefore appears that a similar phenomenon occurs with SP-IPTp where the drug complements naturally acquired antimalarial immunity in order to register a beneficial clinical outcome. While various schools of thought have argued for and against the continued use of SP-IPTp [17, 41, 42], the present findings lend support for its continued use. Moreover, confidence in this position may be further assured when SP is used according to the revised guidelines advocating for more frequent dosing of SP [16]. In practice, this would offset any anticipated reductions in PTP associated with increasing levels of resistance.

Whereas the burden of PM appears to have significantly reduced in Kampala over the years [11], no change in profile is apparent from this study. Histopathological data collected ten years earlier showed prevalence of 64%, 4%, and 32% for active, chronic, and past infection, respectively [10]. The trend in these proportions is comparable to that observed among SP nonusers in this study, that is, 50%, 12.5%, and 37.5% for active, chronic, and past infections, respectively. In addition, despite clear differences in malaria burden between SP users and nonusers, no appreciable differences in birth weights were apparent between the two groups. This may be explained by the fact that most placental infections were of Category 1 (active infection), reflecting infections acquired in late pregnancy. Therefore, the pathological sequelae associated with PM may not have been present long enough to adversely impact foetal nutrition and hence birth weight. It has been suggested that the greatest impact of PM on birth weight occurs when infections are acquired earlier on in pregnancy, especially in the first and second trimesters of gestation [43–45]. Significant LBW would therefore have been anticipated in PM infections classified under Category 2 or Category 3.

The use of placental histology for assessment of the burden of MIP may be considered a major strength of this study. This is a robust and the most sensitive measure for MIP when compared with others such as peripheral, placental, or cord smear microscopy [10, 46]. In addition, placental histology provides a temporal history of malaria over more than half of the gestational period [35]. The requirement for participants in the control arm to provide a spot blood sample for residual drug assay may also be considered another strength of this study. The fact that none of the samples turned out positive suggests that participant selection in the control arm was done reasonably well. This scenario contrasts with a previous study in which up to 25% of pregnant women reporting nonuse of SP were found to have residual drug when tested [34]. Many previous studies reporting ineffectiveness of SP-IPTp have relied on self-reports and ANC records as proof of SP use [14, 15, 19]. Where no effort is made to ascertain actual user compliance, such findings remain questionable, especially coming from resource-constrained settings where unsupervised administration of SP is the norm, contrary to guidelines. As argued elsewhere, the presence of such records does not necessarily translate into intake compliance [21, 22]. Our findings are however limited by the small sample sizes used that may not be adequately powered to detect true differences between the groups. Furthermore, while only 6% of women in the SP group resided outside Kampala and its neighbouring districts, this was not true for the control group, where 21% came from rural upcountry districts. Persons from such areas may be predisposed to higher risks of malaria owing to the rural-urban differences in malaria burden. Ward 5C at Mulago Hospital receives countrywide referrals. Having failed to anticipate and control for this demographic pattern, an element of selection bias in these results cannot be excluded.

Lastly, it is important to note that while 87 women were enrolled in the exposure arm, only 38 (43.7%) returned to deliver at the hospital. Going by previous studies, the phenomenon of high ANC attendance coupled with low skilled attendance at delivery is not new or unique to this study [47–51]. From previous studies, statistics on this phenomenon have ranged from 30 to 60%. Reasons advanced for this practice have included a preference for traditional birth attendants, lack of transport (especially for nighttime onset of labour), and financial constraints as well as the need to perform traditional birth rituals. In some cases, rapidly progressing labour may have compelled women to deliver at home or at an alternative health facility [50, 51].

## 5. Conclusions

Compared to nonuse, histological evidence suggests that two doses of SP-IPTp were better at preventing placental malaria among pregnant women delivering at Mulago Hospital. In view of the limited sample size, these findings call for a larger study that is sufficiently powered in order to ascertain these results. Meanwhile, frequent doses of SP-IPTp as per the revised WHO IPTp policy may offer continued benefit in limiting malaria-related adverse pregnancy outcomes in endemic settings.

## Figures and Tables

**Table 1:** Demographic and obstetric characteristics of SP users and nonusers enrolled in the study.

Characteristic	SP-IPTp users (*n* = 33)	SP-IPTp nonusers (*n* = 33)
Residence in Kampala/Mukono/Wakiso	31 (93.9%)	26 (78.8%)
Male sex of baby	18	20
History of bed-net use	29 (87.9%)	30 (90.9%)
Mean age [years (SD)]	24.24 (3.5)	25.54 (3.7)
Mean birth weight [grams (SD)]	3280 (425)	3050 (436)^*∗*^
Mean gestational age (weeks)	37.6	36.48
Still births	0	1
Twin deliveries	0	2

^*∗*^ *n* = 35, including 2 twin pairs.

**Table 2:** Histopathological profiles of placental malaria among SP users and nonusers delivering at Mulago Hospital, Kampala.

Histopathological classification	SP-IPTp users (*n* = 33)	Proportion of positives (*n* = 4)	SP-IPTp nonusers (*n* = 33)	Proportion of positives (*n* = 16)
Category 0 (no infection)	29 (87.9%)	—	17 (51.5%)	—
Category 1 (active infection)	2 (6.1%)	50%	8 (24.2%)	50.0%
Category 2 (active-chronic)	—	—	2 (6.1%)	12.5%
Category 3 (past-chronic)	2 (6.1%)	50%	6 (18.2%)	37.5%
Total biopsies with malaria footprint	4 (12.1%)	100%	16 (48.5%)	100%
